# Artificial intelligence fracture recognition on computed tomography: review of literature and recommendations

**DOI:** 10.1007/s00068-022-02128-1

**Published:** 2022-10-26

**Authors:** Lente H. M. Dankelman, Sanne Schilstra, Frank F. A. IJpma, Job N. Doornberg, Joost W. Colaris, Michael H. J. Verhofstad, Mathieu M. E. Wijffels, Jasper Prijs, On Behalf of Machine Learning Consortium, Paul Algra, Michel van den Bekerom, Mohit Bhandari, Michiel Bongers, Charles Court-Brown, Anne-Eva Bulstra, Geert Buijze, Sofia Bzovsky, Joost Colaris, Neil Chen, Job Doornberg, Andrew Duckworth, J. Carel Goslings, Max Gordon, Benjamin Gravesteijn, Olivier Groot, Gordon Guyatt, Laurent Hendrickx, Beat Hintermann, Dirk-Jan Hofstee, Frank IJpma, Ruurd Jaarsma, Stein Janssen, Kyle Jeray, Paul Jutte, Aditya Karhade, Lucien Keijser, Gino Kerkhoffs, David Langerhuizen, Jonathan Lans, Wouter Mallee, Matthew Moran, Margaret McQueen, Marjolein Mulders, Rob Nelissen, Miryam Obdeijn, Tarandeep Oberai, Jakub Olczak, Jacobien H. F. Oosterhoff, Brad Petrisor, Rudolf Poolman, Jasper Prijs, David Ring, Paul Tornetta, David Sanders, Joseph Schwab, Emil H. Schemitsch, Niels Schep, Inger Schipper, Bram Schoolmeesters, Joseph Schwab, Marc Swiontkowski, Sheila Sprague, Ewout Steyerberg, Vincent Stirler, Paul Tornetta, Stephen D. Walter, Monique Walenkamp, Mathieu Wijffels, Charlotte Laane

**Affiliations:** 1 Trauma Research Unit, Department of Surgery, Erasmus MC, University Medical Center Rotterdam, P.O. Box 2040, 3000 CA Rotterdam, The Netherlands; 2 Department of Orthopedic Surgery, Groningen University Medical Centre, Groningen, The Netherlands; 3 Department of Surgery, Groningen University Medical Centre, Groningen, The Netherlands; 4 Department of Orthopedic & Trauma Surgery, Flinders Medical Centre, Flinders University, Adelaide, Australia; 5 Department of Orthopedics, Erasmus University Medical Centre, Rotterdam, The Netherlands

**Keywords:** Artificial intelligence, Convolutional neural networks, Fractures, Computed tomography, Orthopedics

## Abstract

**Purpose:**

The use of computed tomography (CT) in fractures is time consuming, challenging and suffers from poor inter-surgeon reliability. Convolutional neural networks (CNNs), a subset of artificial intelligence (AI), may overcome shortcomings and reduce clinical burdens to detect and classify fractures. The aim of this review was to summarize literature on CNNs for the detection and classification of fractures on CT scans, focusing on its accuracy and to evaluate the beneficial role in daily practice.

**Methods:**

Literature search was performed according to the PRISMA statement, and Embase, Medline ALL, Web of Science Core Collection, Cochrane Central Register of Controlled Trials and Google Scholar databases were searched. Studies were eligible when the use of AI for the detection of fractures on CT scans was described. Quality assessment was done with a modified version of the methodologic index for nonrandomized studies (MINORS), with a seven-item checklist. Performance of AI was defined as accuracy, F1-score and area under the curve (AUC).

**Results:**

Of the 1140 identified studies, 17 were included. Accuracy ranged from 69 to 99%, the F1-score ranged from 0.35 to 0.94 and the AUC, ranging from 0.77 to 0.95. Based on ten studies, CNN showed a similar or improved diagnostic accuracy in addition to clinical evaluation only.

**Conclusions:**

CNNs are applicable for the detection and classification fractures on CT scans. This can improve automated and clinician-aided diagnostics. Further research should focus on the additional value of CNN used for CT scans in daily clinics.

**Supplementary Information:**

The online version contains supplementary material available at 10.1007/s00068-022-02128-1.

## Introduction

The use of computed tomography (CT) in trauma care is substantially increasing. In the Netherlands, over 2 million CT scans were made in 2019 and this number increases each year [1]. Total-body CTs are increasingly used in acute trauma settings and can be more cost-effective than standard radiological imaging [2]. Increased use of imaging strains radiologists, to the point of creating a shortage of radiologist in hospitals [3]. Examining CT scans and radiographs to detect and classify fractures can be time consuming, challenging, and poor inter-observer variability among radiologists and (experienced) clinicians can be substantial [3]. Artificial intelligence (AI) could play a big role optimizing workflows in the acute setting and allow clinicians to spend their time more effectively.

AI can execute different tasks, ranging from searching the web to self-driving cars—tasks that until a few years ago could only be performed by humans. Deep learning (DL) is a subset of machine learning (ML) that uses mainly convolutional neural networks (CNNs) [4]. CNNs are combinations of artificial neuron layers with different units. These units operate like neurons of our brain [3]. CNNs can learn to recognize discriminative features from data and assign importance to various aspects in the image and to differentiate one from another. An example of data used to train an ankle fracture CT CNN is presented in Supplemental Video 1. While most earlier AI methods have led to applications with subhuman performance, recent CNNs are able to match and even surpass the capacity of humans detecting certain fractures on radiographs, focusing on isolated fracture types per model [5–9]. The strength of computers is their ability to evaluate a vast number of examinations rapidly, consistently and without exhaustion.

When clinicians are aided by DL-based automatic fracture detection algorithms, the accuracy of clinical diagnosis might improve and time to diagnosis reduced, which can be useful in, among others, an emergency setting. Various studies have successfully applied CNNs to detect fractures of various body parts on radiographs [5–9]. The results in detecting and classifying fractures on radiographs by CNNs are promising. However, only a few studies have developed CNNs for the detection of fractures on CT scans. Therefore, we conducted this systematic review to give an overview of studies using AI with CT scans to detect or classify fractures. The aim of this study was to answer the following questions: 1) What is the accuracy of a CNN in detecting fractures on CT scans? 2) Does the use of CNNs with CT scans improve the diagnostic performance of clinicians?

## Materials and methods

### Article selection, quality assessment and data extraction

**Fig. 1:**
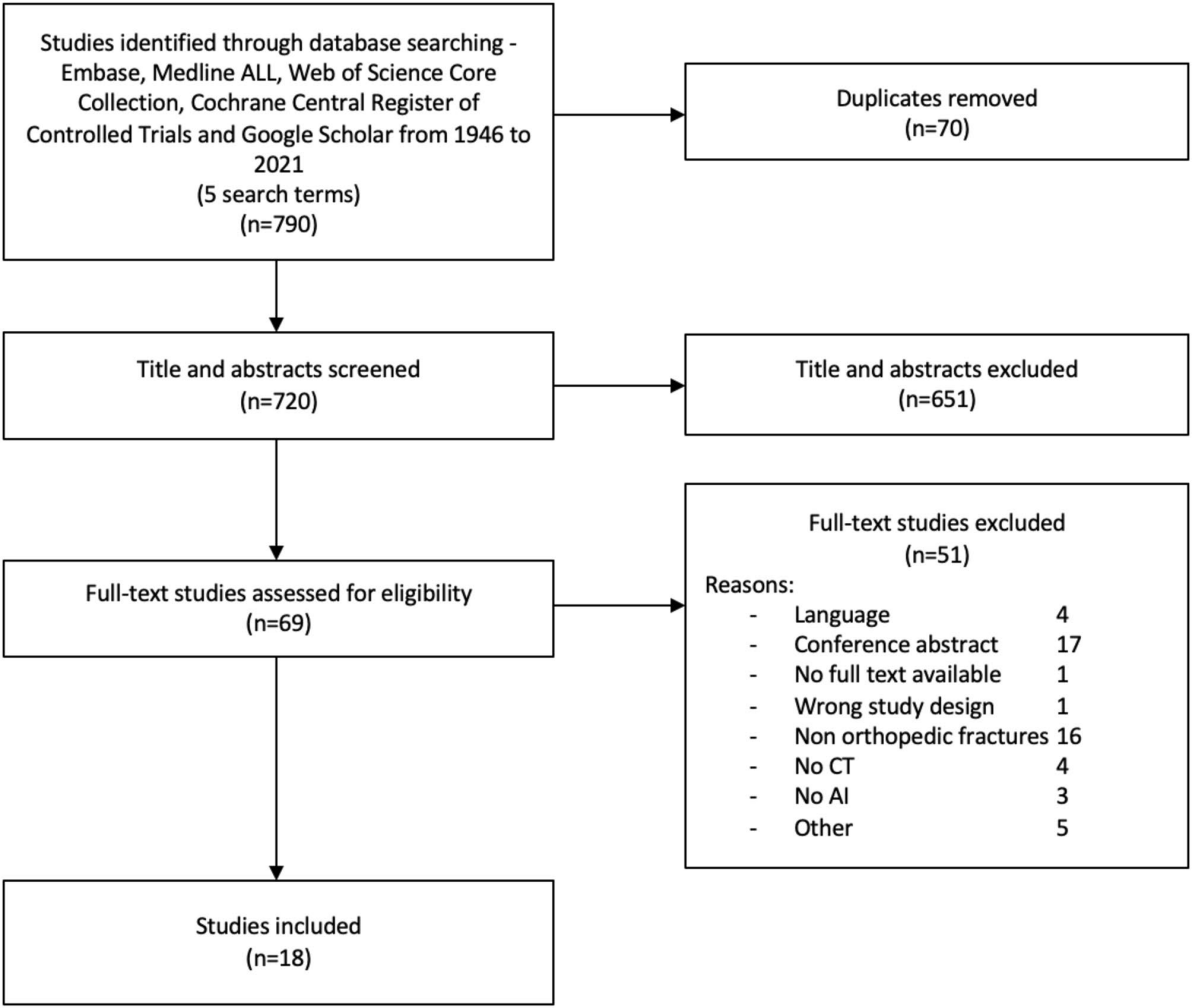
PRISMA flow chart

A systematic literature search was performed according to the PRISMA statement [10] (Fig. 1) and conducted in the following libraries: Embase, Medline ALL, Web of Science Core Collection, Cochrane Central Register of Controlled Trials and Google Scholar. The search strategy was formulated together with a librarian (see appendix 1).

All published articles were searched. After removing duplicates, titles and abstracts of the potentially eligible articles were independently screened by two reviewers (LD, SS). Subsequently, full-text screening was performed using the predefined criteria to check eligibility. If the conclusion was inconsistent, a third reviewer was consulted (JP). Articles met the inclusion criteria if AI was used to detect fractures on CT scans in an orthopedic trauma setting. The defined exclusion criteria were: review articles or letters, conference abstracts, technique papers, studies using robots, animal and cadaveric studies, non-orthopedic fractures and studies not published in English or Dutch. Covidence (Veritas Health Innovation, Melbourne, Australia) was used for the screening process and full-text review.

The quality of all included articles was assessed by two independent reviewers (LD, SS). In case of a disagreement, a third reviewer was consulted (JP). For the quality assessment, a modified version of the methodologic index for nonrandomized studies (MINORS) instrument was used, including the following items: disclosure, study aim, input features, ground truth, dataset distribution, performance metric and AI model (Table 1). Studies with low scores on three or more items were excluded. Standardized forms were used to extract and record data (Microsoft Excel Version 16.21; Microsoft Inc, Redmond, WA, USA).

**Table 1:** Quality assessment according to MINORS criteria

Author, year	Study type	Disclosure	Study aim	Input feature	Ground truth	External validation method	Performance metric	AI model
Castro-Zunti et al [17]	Classification	1	1	1	1	1	1	1
Dreizin et al [18]	Classification	1	1	1	1	1	1	1
Hu et al [13]	Detection/classification	1	1	0	1	1	1	1
Jin et al [27]	Detection	1	1	1	1	1	1	1
Kaiume et al [19]	Detection	1	1	1	1	1	1	1
Meng et al [20]	Detection/classification	1	1	1	1	1	1	1
Pranata et al [15]	Detection/classification	1	1	1	0	1	1	1
Raghavendra et al [16]	Detection	1	1	1	0	1	1	1
Roth et al [11]	Detection	0	1	0	0	1	1	1
Small et al [14]	Detection	1	1	0	1	1	1	1
Ukai wet al [21]	Detection	1	1	1	1	1	1	1
Voter et al [28]	Detection	1	1	1	1	1	1	1
Weikert et al [22]	Detection	1	1	1	1	1	1	1
Yacoub et al [23]	Detection	1	1	1	1	1	1	1
Yamamoto et al [12]	Detection	0	1	1	1	1	1	1
Yoon et al [24]	Detection/classification	1	1	1	1	1	1	1
Zhou et al [25]	Detection/classification	1	1	1	1	1	1	1
Zhou et al [26]	Detection/classification	1	1	1	1	1	1	1

### Outcome measures

In this study, the primary outcome was performance of the CNNs used, measured by their accuracy, F1-scores and area under the curve (AUC). Seventeen studies met the inclusion criteria and were used to answer this research question. To answer the secondary question in this study, ten studies comparing performance of the CNN to performance clinicians were used.

The data points collected from each study were: author, year of publication, anatomical location of the fracture, AI models used (type), imaging direction of CT slices, output classes, ground truth label assignment, number of patients and performance metric (e.g., accuracy, AUC curve) (Table 2).

**Table 2:** Discription of studies

Author, year	Anatomical location	AI models used (Type)	Imaging direction of CT slices	Output classes	Ground truth label assignment	Number of fractures	Performance metric	Performance	Comparison CNN vs. radiologist
Castro-Zunti et al., 2021 [17]	Ribs	CNN (InceptionV3)	Axial	2, 3 subclasses	2 radiologists	*N* = 612^a^	Accuracy AUC	3 classes: 96.00% Binary: 97.76% Binary: 94.7% (95% CI 94.1–95.3)^c^	Yes
Dreizin et al., 2021 [18]	Pelvic	CNN (ResNetXt-50 + LSTM)	Axial, coronal, sagittal	2	3 radiologists	*N* = 373 ^a^	Accuracy F1 score	85%; 74% (discriminating translational and rotational instability respectively) 0.63; 0.77	Yes
Hu et al., 2021 [13]	Ribs	CNN (SGANet)	Axial	2	2 doctors	*N* = 398	Accuracy; F1 score	82.54% 0.7843	Yes
Jin et al., 2020 [27]	Ribs	DL (FracNet)	Axial	2	2 radiologists and 2 junior radiologists	*N* = 7473	(Sensitivity)	92.9%	Yes
Kaiume et al., 2021 [19]	Ribs	CNN (DenseNet + SSD)	Axial	2	2 radiologists	*N* = 256	F1 score;	0.711	No
Meng et al., 2021 [20]	Ribs	CNN (VRB-Net)	Axial	2, 4 subclasses	2 senior radiologists	*N* = 861	Accuracy F1 score	86.3% 0.940	Yes
Pranata et al., 2019 [15]	Calcaneus	CNN (ResNet; VGG)	Coronal, sagittal, axial	2	N.A	*N* = 1931	Accuracy	ResNet: 80–98% VGG: 92–98%	No
Raghavendra et al., 2018 [16]	Thoracolumbar	CNN	Sagittal	2	N.A	*N* = 700	Accuracy	99.1% max; 96.51% average	No
Small et al., 2021 [16]	Spine	CNN (ResNet)	Axial, coronal, sagittal	2	2 neuroradiologists	*N* = 143	Accuracy	92% (95% CI, 90–94%)	Yes
Ukai et al., 2021 [21]	Pelvic	CNN (YOLOv3)	Axial, coronal, sagittal (3D)	3	Orthopedic surgeons	*N* = 389	F1-score AUC	0.853 0.824	No
Voter et al., 2021 [28]	CSFx	AI DSS (Aidoc)	Axial	2	Neuroradiologist; radiology	*N* = 173	F1-score^b^	0.453*	No
Weikert et al., 2020 [22]	Ribs	CNN (ResNet)	Axial	2, 3 subclasses	Written CT reports approved by a board certified radiologist	*N* = 159	Accuracy F1 score	90.2% (95% CI 87.3–92.6) 0.85	Yes
Yacoub et al., 2021 [23]	VCF	CNN (AI-Rad Companion)	Sagittal	2	2 radiologists	*N* = 100 ^a^	AUC F1-score^b^	0.82 (95% CI 0.73–0.89) 0.352^b^	Yes
Yamamoto et al., 2020 [12]	Pelvic	CNN (VGG-16)	Sagittal, axial, coronal (3D)	2	4 residents and 5 orthopaedic specialists	*N* = 103 ^a^	Accuracy F1-score^b^	69.4% 0.578^b^	No
Yoon et al., 2020 [24]	Femur	CNN (Faster R-CNN)	Sagittal, axial, coronal (3D)	2 groups, 10 subclasses	Orthopedic surgeons	*N* = 3343	Accuracy	Per class: 2: 97% $\pm 0.02$ 3: 95% $\pm 0.02$ 4: 94% $\pm 0.01$ 7: 92% $\pm 0.01$ 10: 90%$\pm 0.02$	No
Zhou et al., 2021 [25]	Ribs	CNN (Faster R-CNN, ResNet-101)	Axial	3	2 musculoskeletal radiologists, 2 senior radiologists, thoracic surgeon	*N* = 4215	F1 score Accuracy AUC	Model I vs. Model I/T 1: 0.814 vs. 0.875 2: 0.816 vs. 0.847 3: 0.378 vs. 0.839 1: 78.8% vs. 85.2% 2: 81.3% vs. 90.4% 3: 73.9% vs. 88.5% 1: 83.6% vs. 90.7% 2: 88.7% vs. 94.2% 3: 77.0% vs. 90.5%	Yes
Zhou et al., 2020 [26]	Ribs	CNN (Faster R-CNN, YOLOv3)	Axial	3	2 musculoskeletal radiologists, 2 senior radiologists, thoracic surgeon	*N* = 1079^a^	F1 score	1: 0.849 2: 0.856 3: 0.770 Mean: 0.825	Yes

*AI* artificial intelligence, *DSS* decision support systems, *CNN* Convolutional Neural Networks, *DL* deel learning, *AUC* Area Under the Curve, *LSTM* a long short-term memory network, *DenseNet* Densely connected convolutional Network, *SSD* single shot multibox detector, *ResNet* Residual network, *SGAnet* slice grouping and aggregation network, *VGG* Visual geometry group, *VRB-net* V-net, ResNey and Bottleneck ResNet Network, *YOLOv3* You Only Look Once, version 3

^a^Number of fractures not given, number of patients stated

^b^F1-score calculated with

^c^AUC score given in percentages

Output classes included fracture detection (i.e., fracture yes/no) and/or classification (i.e., OA/OTA classification). All studies described the detection of fractures by the CNN, and seven studies also performed fracture classification.

Studies used accuracy, F1-score and AUC to measure the performance of the model. The F1-score (2*((precision*recall)/(precision + recall)) is the harmonic mean of the precision (positive predictive value) and recall (sensitivity) of the test, where it requires both to be high for a favorable F1-score. The highest possible value is 1.0, indicating a perfect precision and recall, and the lowest possible value is 0. If not assessed, the F1-score was calculated when precision and recall were stated. The area under the curve (AUC) is a score to measure the ability of a classifier to distinguish between classes. The score lies between 0.5 (a classifier equal to that of chance) and 1 (an excellent classifier). Where possible, accuracy and/or F1-scores were calculated to facilitate comparison between studies.

### Quality appraisal

The modified MINORS tool included the following items: disclosure, study aim, input feature, ground truth, dataset distribution and performance metric (Table 1). Disclosure was reported in all but two studies [11, 12]. All studies clearly stated their study aim, model used and how performance was measured. The input feature was not clearly specified in three studies [11, 13, 14]. These studies did not mention what the inclusion and exclusion criteria were. Three studies did not specify the ground truth (the reference standard used in AI) [11, 15, 16]. One study was excluded after the quality assessment, because it scored too low on three items: disclosure, input feature, and ground truth [11].

## Results

### Included studies

The search yielded a total of 1140 articles. After duplicate removal, 720 abstracts were screened. Sixty-nine studies were selected for full-text screening, of which eighteen remained. No new eligible studies were identified through screening the reference lists. One study was excluded after quality assessment, because the risk of bias was deemed too high due to unclear reporting of disclosure, input feature and ground truth [11]. Seventeen studies were used for analysis.

### Description of studies

All seventeen studies used a CNN to detect and /or classify fractures on CT scans [12–28]. Eight studies addressed detection of rib fractures [13, 17, 19, 20, 22, 25–27], three studies the performance for detection [12, 21] and classification [18] of pelvic fractures, four for detection of spine fractures [14, 16, 23, 28], one for detection and classification of femur fractures [24] and one of calcaneal fractures [15]. Fourteen studies used two output classes (fracture yes/no). One study on spine fractures used three output classes: completely displaced, incompletely displaced and compression fracture [14]. In addition, two studies used fresh, healing and old fracture as output classes [25, 26]. In 12 studies, the ground truth for diagnosis and classification of the fractures was the conclusion of two or more experts, who interpreted the CT scans independently [12–14, 17–20, 23, 25–28]. One study used radiology reports from routine care as ground truth [22]. Two studies did not specify how many experts provided the ground truth [21, 24]. Thereby, two studies did not report the ground truth [15, 16]. The number of patients included in the studies ranged from 39 [19] to 8529 [20] fractures.

### Primary outcome: the performance of CNN

The performance was defined in various ways among studies. Accuracy on detection and/or classification was measured in eleven studies [12–18, 20, 22, 24, 25], ranging from 69.4% [12] to 99.1% [16]. Eight studies used the F1-score to assess performance instead: in two the F1-score was assessed for the classification of healing status [25, 26], in one for displacement [21], and in five [13, 18–20, 22] for the detection of fractures. Additionally, we calculated the F1-scores in three studies [12, 23, 28] to facilitate comparison. F1-scores ranged from 0.35 in Yacoub et al. [23] to 0.94 in Meng et al. [20]. Four studies reported the AUC as a performance metric [17, 21, 23, 25], ranging from 0.770 [25] to 0.947 [17]. Zhou et al. [25] reported the AUC on classification of challenging fractures compared to the other three studies with more simple fracture detection. One study just reported a sensitivity of 92.9% [27].

In Castro-Zunti et al. [17], the accuracy and AUC scores of four different AI models were compared for 612 patients. They found that the CNN model InceptionV3 achieved the highest average accuracy of 96%, when the CT slices were divided into three classes (acute, old (healed) and normal (non-fractured). In Yoon et al. [24], the data were divided into ten classes (based on the AO/OTA classification [29]) and the accuracy of the different numbers of output classes was reported for 85 patients. Binary classification (no fracture vs fracture) achieved the highest accuracy of 97%. When the data were divided into more classes (AO/OTA classification [29]), the accuracy decreased to the lowest value of 90% for ten classes, as compared to the ground truth by orthopedic surgeons. Dreizin et al. [18] reported the superiority of translational instabilities (85%) over rotational ones (74%) on the accuracy and F1-score of their model [18] for 373 patients. Zhou et al. [25] reported improved performance on 1020 patients using CTs combined with patient information compared (accuracy for three different models: 85.2%, 90.4% and 88.5%) to just CTs alone (accuracy for three different models: 78.8%, 81.3% and 73.9%) [25]. In another—earlier—study, Zhou et al. [26] reported that the mean F1-score of healing rib fractures was the highest and of old fractures the lowest (0.856 vs. 0.770).

**Fig. 2:**
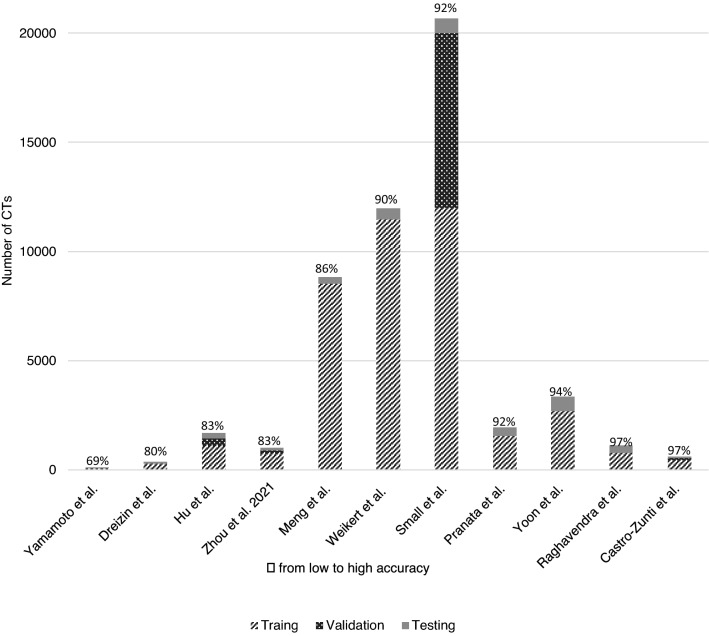
Correlation between accuracy and total number of CTs

In Fig. 2, the amount CTs for training, validation and testing are plotted against the accuracy, with increasing accuracy from left to right. The study with the most CTs reported an average accuracy of 92% [14]. The highest accuracy of 97% was reported in a study [17] with only 612 CTs.

In summary, the reported outcomes on accuracy (ranging from 69.4 to 99.1%), the F1-score (from 0.35 to 0.94), the AUC (from 0.770 to 0.947) and the sensitivity (92.9%) were assessed on different classifications, CNN models and training, validation and testing sets across the included studies.

### Secondary outcome: CNN and clinicians

Ten out of seventeen studies compared a CNN model to the diagnostic performance of radiologists [14, 17, 22, 23, 25–27] or radiology reports [13, 22, 23]. Seven [14, 17, 22, 23, 25–27] out of these ten studies compared the sensitivity of a CNN model to radiologists. In three studies [17, 26, 27], the CNN model solely or as an additional CNN model resulted in a higher sensitivity compared to the radiologist alone. Three studies showed a similar sensitivity for CNN and radiologist, [22, 23, 25] and one [14] showed a decrease in sensitivity with CNN. Four studies reported a significant reduction in time to diagnosis when a radiologist was aided by a CNN [20, 25–27].

Two out of ten studies compared the accuracy of CNN vs clinicians [18, 20]. In Meng et al. [20], junior radiologists significantly improved their accuracy when assisted by a CNN for detection and classification of fractures. Experienced radiologists showed similar improvement [20]. In Dreizin et al. [18], the model was equivalent in accuracy compared to radiologists. One study showed that when CNN is combined with clinical reports, the number of missed diagnoses is reduced by 88% [13].

In summary, the four studies [13, 20, 26, 27] that reported the performance of a CNN as an aid for the radiologist showed that CNN increases the performance of detection and classification of fractures. Twelve [13, 15–20, 22, 24–27] out of seventeen studies concluded that the use of a CNN improved or could improve clinical care. In the remaining five studies, three studies [14, 21, 28] recommend CNN as a second-stage interpretation to assist radiologists, in one performance was inferior to clinical radiology reports [23] and lastly, one did not report on improvement [12].

## Discussion

In this systematic review, the results of several studies using AI for fracture detection and classification—in particular convolutional neural networks (CNNs)—were analyzed. The included studies reveal that CNNs show good performance in detecting and classifying various fractures on CT scans. The use of CNNs may add value and efficiency to several components of the skeletal imaging workflow in trauma care. The overall conclusion in most of studies was that CNNs are applicable in aiding clinicians, by reducing both time to diagnosis and number of missed diagnoses while improving the diagnostic performance. In addition, CNNs have proven to be very consistent, in contrast to the high inter-observer variability among radiologists and surgeons, when interpreting CT scans [3]. Due to the scarcity of studies reviewing the place of CNNs in trauma CT imaging, the search strategy was very broad, and various libraries were queried. In addition, this study looks at the comparison of CNN versus clinicians or CNN as an assistant for clinicians.

This study should be interpreted in light of strengths and weaknesses. First, comparability of the studies is limited, because some fractures may be easier to detect, have different characteristics, and are in different surrounding anatomical structures than others. However, the results of the studies show comparable performances across the board and this heterogenicity did not affect answering our research questions. Secondly, different definitions for the ground truth were used among the various studies. For example, ground truth labels might be determined by various numbers of radiologists with different levels of expertise. An important note is that all these reference standards are subject to human biases. Lastly, to date, only a small number of studies have investigated the use of AI for fracture detection on CT scans, in limited patient group sizes. This may overestimate the potential benefit of AI, and therefore, future research should overcome this shortcoming. In addition, for the use of CNN models in daily practice, these models need to be further developed, with greater training and testing sets, external validation and prospective validation. However, if the beneficial effect of AI in fracture diagnosing and treatment results in improvement, this might impede extensive changes for the daily clinic. Strengths include the search of multiple databases, the use of a modified MINORS that included CNN-specific factors such as the input feature, ground truth, dataset distribution and performance metric. Future studies investigating AI on CTs for fracture detection and classification should include a wide data base of training, validation and testing sets, report demographic and diagnostic performance metrics, external validation of the CNN model [30] and the investigation of more common fractures (for example, wrist and ankle).

In general, for CNNs, it is assumed that the larger the dataset, the higher the performance. Training with a small dataset is a major cause of overfitting and does not lead to suitable generalization of performance. Due to the heterogeneity of the studies, straightforward conclusions for the recommended size of datasets cannot be drawn. However, a clear correlation for all fractures sites between accuracy and data size, with some studies reaching perfect accuracy with small datasets of less than 1200 CT scans, seems to be lacking. Taking this in consideration, in combination with the limited time of experts to provide high-quality labels, we recommend a stepwise approach of small dataset that increases in increments until adequate performance, or plateau is reached.

Most studies used the same base CNN architectures. Five studies used ResNet [14, 15, 18, 22, 25]. They showed a similar accuracy, while investigating different anatomical locations. Two studies used YOLOv3 and both showed similar F1-scores [21, 26]. Two studies used the CNN model VVG-16 [12, 15]. The accuracy measured in these studies was divergent. Pranata et al. [15] presented a very accurate CNN model for detection of calcaneal fractures, while the accuracy found for detection of pelvic fractures [12] was significantly lower. A reason for this difference could be the group size of both studies; 1931 calcaneal fractures vs. 103 pelvic fractures. Furthermore, the stability of the pelvis is based both on bony and/or ligamentous injury, a much more challenging task compared to finding cortical fractures.

RestNet (or a modified version) was the most used CNN network, with reported accuracies between 73 and 98%. The best-performing model was reported by Raghavendra et al. [16] that showed an average accuracy of 96.51%. This model was developed by the authors, however, without external validation which warrants some caution in interpretation of the results [30]. Less than half (6/17) of all studies reported the use of an external validation. To implement in clinical practice, external validation of CNN models is crucial to explore transportability and bias [30] and will be the topic of future studies.

Other fields are ahead of orthopedics with regard to the use of CNNs as computer-aided detection. CNNs have been reported in oncology for: the classification of biopsy-proven masses and normal tissue on mammograms [31], classification of skin cancer [32] and the automated detection of pathological mediastinal lymph nodes in lung cancer [33]. CNNs have been shown to improve diagnostic performance in detection of lung nodes and coronary artery calcium on CTs in lung cancer screening [34]. The use of CNNs in fracture detection and classification is only following in the footsteps of much further developments in other specialties.

In conclusion, CNNs can detect fractures and important fracture characteristics on CT scans, which may be used to guide treatment and optimize diagnosis of fractures. In addition, computers can evaluate a vast number of examinations rapidly, consistently and without exhaustion. If CNNs are trained well, using at least multiple experts to provide the ground truth, this could reduce the inter-observer variability plaguing daily practice, and be a valuable application in a trauma setting by reducing time to diagnosis. Further research is needed to explore strengths and weaknesses of CNNs in an acute trauma setting.

### Electronic supplementary material

Below is the link to the electronic supplementary material.

Supplementary file1 (MP4 36577 KB)

## Appendix: 1. Search

### Narrative review

**Table:**

Database searched	via	Years of coverage	Records	Records after duplicates removed
Embase	Embase.com	1971—Present	440	430
Medline ALL	Ovid	1946—Present	272	105
Web of Science Core Collection*	Web of Knowledge	1975—Present	304	134
Cochrane Central Register of Controlled Trials	Wiley	1992—Present	24	13
Other sources: Google Scholar (100 top-ranked)			100	39
Total			1140	721

*Science Citation Index Expanded (1975-present); Social Sciences Citation Index (1975-present); Arts & Humanities Citation Index (1975-present); Conference Proceedings Citation Index- Science (1990-present); Conference Proceedings Citation Index- Social Science & Humanities (1990-present); Emerging Sources Citation Index (2015-present)

### Embase 440

('fracture'/exp OR ('injury'/de AND 'orthopedics'/de) OR ((fracture* NOT (root-fractur* OR dental)) OR ((traum* OR injury OR injuries) AND orthop*) OR ((broken) NEAR/6 (bone*))):ab,ti,kw) AND ('x-ray computed tomography'/exp OR 'computed tomography scanner'/exp OR 'radiomics'/de OR 'bone radiography'/de OR 'computer assisted tomography'/de OR 'radiography'/de OR 'joint radiography'/de OR (((X-ray) NEAR/3 (tomograph*)) OR CT OR CT$imag* OR CT$scan* OR ((compute*) NEAR/3 (tomograph*)) OR radiomic* OR radiograph* OR arthrograph*):ab,ti,kw) AND ('convolutional neural network'/de OR 'machine learning'/exp OR 'artificial intelligence'/exp OR (((neural*) NEAR/3 (network*)) OR CNN OR ((machine* OR deep*) NEAR/3 (learn*)) OR ((artific* OR machin*) NEAR/3 (intelligen*)) OR support-vector*):ab,ti,kw) NOT ((animal/exp OR animal*:de OR nonhuman/de) NOT ('human'/exp)).

### Medline 272

(exp Fractures, Bone/ OR (Wounds and Injuries/ AND Orthopedics/) OR ((fracture* NOT (root-fractur* OR dental)) OR ((traum* OR injury OR injuries) AND orthop*) OR ((broken) ADJ6 (bone*))).ab,ti,kf.) AND (exp Tomography, X-Ray Computed/ OR Tomography Scanners, X-Ray Computed/ OR Radiography/ OR Arthrography/ OR (((X-ray) ADJ3 (tomograph*)) OR CT OR CT$imag* OR CT$scan* OR ((compute*) ADJ3 (tomograph*)) OR radiomic* OR radiograph* OR arthrograph*).ab,ti,kf.) AND (exp Artificial Intelligence/ OR (((neural*) ADJ3 (network*)) OR CNN OR ((machine* OR deep*) ADJ3 (learn*)) OR ((artific* OR machin*) ADJ3 (intelligen*)) OR support-vector*).ab,ti,kf.) NOT (exp Animals/ NOT Humans/).

### Cochrane 24

(((fracture* NOT (root-fractur* OR dental)) OR ((traum* OR injury OR injuries) AND orthop*) OR ((broken) NEAR/6 (bone*))):ab,ti,kw) AND ((((X-ray) NEAR/3 (tomograph*)) OR CT OR CT-imag* OR CT-scan* OR ((compute*) NEAR/3 (tomograph*)) OR radiomic* OR radiograph* OR arthrograph*):ab,ti,kw) AND ((((neural*) NEAR/3 (network*)) OR CNN OR ((machine* OR deep*) NEAR/3 (learn*)) OR ((artific* OR machin*) NEAR/3 (intelligen*)) OR support-vector*):ab,ti,kw).

### Web of Science 304

TS = ((((fracture* NOT (root-fractur* OR dental)) OR ((traum* OR injury OR injuries) AND orthop*) OR ((broken) NEAR/5 (bone*)))) AND ((((X-ray) NEAR/2 (tomograph*)) OR CT OR CT-imag* OR CT-scan* OR ((compute*) NEAR/2 (tomograph*)) OR radiomic* OR radiograph* OR arthrograph*)) AND ((((neural*) NEAR/2 (network*)) OR CNN OR ((machine* OR deep*) NEAR/2 (learn*)) OR ((artific* OR machin*) NEAR/2 (intelligen*)) OR support-vector*)) NOT ((animal* OR rat OR rats OR mouse OR mice OR murine OR dog OR dogs OR canine OR cat OR cats OR feline OR rabbit OR cow OR cows OR bovine OR rodent* OR sheep OR ovine OR pig OR swine OR porcine OR veterinar* OR chick* OR zebrafish* OR baboon* OR nonhuman* OR primate* OR cattle* OR goose OR geese OR duck OR macaque* OR avian* OR bird* OR fish*) NOT (human* OR patient* OR women OR woman OR men OR man))).

### Google Scholar

fracture ‘X-ray tomography’|CT|’CT-image’|’CT-scan’|’computed tomography’|radiography ‘neural network’|CNN|’machine|deep learning’|’artificial|machine intelligence’|’support-vector’ -root -dental.
